# Long-Term Effects of Continuous Positive Airway Pressure (CPAP) Therapy on Obesity and Cardiovascular Comorbidities in Patients with Obstructive Sleep Apnea and Resistant Hypertension—An Observational Study

**DOI:** 10.3390/jcm9092802

**Published:** 2020-08-30

**Authors:** Roxana Pleava, Stefan Mihaicuta, Costela Lacrimioara Serban, Carmen Ardelean, Iosif Marincu, Dan Gaita, Stefan Frent

**Affiliations:** 1 Department of Cardiology, “Victor Babes” University of Medicine and Pharmacy, Eftimie Murgu Sq. no. 2, 300041 Timisoara, Romania; roxana.pleava@gmail.com (R.P.); dgaita@cardiologie.ro (D.G.); 2 Department of Pulmonology, “Victor Babes” University of Medicine and Pharmacy, Eftimie Murgu Sq. no. 2, 300041 Timisoara, Romania; frentz.stefan@umft.ro; 3 Cardioprevent Foundation, 300298 Timisoara, Romania; carmenardelean79@yahoo.com; 4 Department of Functional Sciences, “Victor Babes” University of Medicine and Pharmacy, Eftimie Murgu Sq. no. 2, 300041 Timisoara, Romania; costela.serban@umft.ro; 5 Department of Infectious Diseases, “Victor Babes” University of Medicine and Pharmacy, Eftimie Murgu Sq. no. 2, 300041 Timisoara, Romania; imarincu@umft.ro

**Keywords:** sleep apnea, resistant hypertension, body-mass index, CPAP therapy

## Abstract

Background: We sought to investigate whether long-term continuous positive airway pressure (CPAP) treatment in patients with obstructive sleep apnea (OSA) and resistant hypertension (RHTN) could attenuate the cardiovascular disease risk by lowering their body-mass index (BMI). Methods: This was a long-term observational study of RHTN patients diagnosed with OSA. Patients were evaluated with polysomnography initially and after a mean follow-up period of four years. The patients were divided into two groups based on their compliance to CPAP therapy. Results: 33 patients (aged 54.67 ± 7.5, 18 men, 54.5%) were included in the study, of which 12 were compliant to CPAP therapy. A significant reduction in BMI at follow-up was noted in patients compliant to CPAP therapy (1.4 ± 3.5 vs. −1.6 ± 2.5, *p* = 0.006). We also noted a large effect size reduction in abdominal circumference at follow-up in the CPAP group. At follow-up evaluation, the mean heart rate (b/min) was lower in the CPAP group (58.6 ± 9.5 vs. 67.8 ± 7.8), while arrhythmia prevalence increased between initial (28.6%) and follow-up (42.9%) evaluation with an intermediate effect size in non-compliant patients. Conclusions: In our cohort of OSA patients with RHTN, long-term adherence to CPAP therapy was associated with weight loss and improvement in cardiac rhythm outcomes.

## 1. Introduction

Resistant hypertension (RHTN) is defined as blood pressure that remains above 140/90 mmHg despite the use of at least three antihypertensive medications of different classes at the best tolerated doses [1]. While most patients fail to reach the targeted blood pressure values despite treatment and lifestyle adjustment, it is estimated that up to 10% of hypertensive patients have RHTN [2].

Obstructive sleep apnea (OSA) is a common sleep disorder with a prevalence of up to 21% in the general population and a significant increase with age [3]. It is defined by recurrent episodes of upper airway obstruction that determines sleep fragmentation, intermittent hypoxia and increased sympathetic activity, further triggering intermediate mechanisms, including elevated blood pressure with a non-dipper blood pressure pattern [4]. The prevalence of OSA is particularly elevated in patients with RHTN compared to the general hypertensive cohort. One of the pathways of OSA induced hypertension includes hyperaldosteronism, as it is common both in RHTN and OSA patients [5]. The mechanism is not yet fully elucidated, but it may be related to aldosterone-induced fluid retention, especially in the neck area that leads to upper airway resistance [6]. Alongside fluid overload, sympathetic hyperactivity is considered to be an important contributor to RHTN. Combined with age, obesity and chronic kidney disease, both hyperaldosteronism and fluid overload impact the renin-angiotensin aldosterone axis [7]. The more severe the OSA, the greater is the impact on the mechanisms above, leading to an increase in blood pressure.

The prevalence of obesity has increased in recent years, especially in developing countries [8], with a rate varying from 39% to 98% [9]. Obese patients have a higher risk of OSA as obesity promotes soft tissue enlargement surrounding the airway, thus narrowing the pharyngeal passage. Fat deposits in the tongue, soft palate and uvula can also contribute to the genesis of OSA [10]. The treatment of hypertension in obese patients can be very challenging due to the high prevalence of RHTN and other frequently associated comorbidities [11]. There is a multitude of pathophysiological mechanisms that may play a role in obesity-related hypertension including oxidative stress, insulin resistance, adiponectin and leptin interaction, and the sympathetic nervous system activity [12]. There is bidirectional interplay between all these mechanisms, finally leading to a maintained state of inflammation in the body [13]. Hypertension and obesity are also associated with a higher cardiovascular risk by promoting endothelial dysfunction and cardiac remodeling [14].

Lifestyle changes likely leading to weight loss can also reduce the risk of cardiovascular events in obese patients, and should be recommended to all patients with OSA, as long as it is combined with other therapies [15]. Evaluation and treatment of OSA is critical as poor sleep quality and a fragmented sleep due to reduction of rapid eye movement (REM) sleep duration can promote positive energy balance and eventually weight gain through higher insulin levels and lower glucagon-like peptide 1 (GLP-1) [16]. Looking further into energetic metabolism, even short time modified sleep architecture with decreased REM sleep was associated with reduced resting metabolic rate and increased intake of energy, carbohydrates and fat [17].

It has been proven that CPAP therapy contributes to weight control in OSA patients through several mechanisms. Probably the most important one is the improvement in leptin resistance and the altering of ghrelin plasma levels, thus improving the hunger and appetite control [18]. Second, it may improve daytime vigilance allowing patients to be more active during the day [19]. Although CPAP treatment is a long-term therapy, there are few studies that show an effect of CPAP use in achieving weight loss [20]. Recurrent problems are the short follow-up period, low adherence to therapy and small patient numbers [21].

It has been suggested that using CPAP treatment in patients with OSA and RHTN may reduce the cardiovascular risk by lowering aldosterone excess [5]. There is a positive and linear correlation between the hours of CPAP use and blood pressure reduction, as CPAP treatment increases the percentage of normal nocturnal blood pressure dipper pattern [22].

Several studies have shown the positive effect that CPAP therapy has on blood pressure and, especially in those with RHTN, in lowering systolic and nocturnal diastolic pressure [23,24]. The observed reduction in blood pressure is usually mild; however even small changes can contribute to a significant reduction in cardiovascular risk. It is thus recommended that all patients with RHTN be tested for OSA, even in the absence of typical symptoms [15].

Positive airway pressure therapy was not found to systematically change BMI in the European Sleep Apnea Database cohort (*n* = 1415, 77% male, mean age 54 ± 1 years, BMI 31.7 ± 6.4 kg/m^2^, apnea-hypopnea index 37 ± 24 n/h, Epworth Sleepiness Scale (ESS) 10.2 ± 5.0), but the response was heterogeneous. In the obese subgroup, BMI was reduced after positive airway pressure treatment (−0.3 [−0.5 to −0.1] kg/m^2^, *p* < 0.05) mainly in patients with a strong reduction in ESS [25].

We hypothesized that long-term CPAP use in patients with RHTN may have a role in reducing BMI, thus lowering the cardiovascular risk.

## 2. Materials and Methods

We conducted an observational, case-control, single centered study at “Victor Babes” Infectious Disease and Pulmonology Hospital, Timisoara. Patients were recruited from those referred to the sleep laboratory with a clinical suspicion of sleep apnea. Written informed consent was obtained from all patients and the study protocol was approved by the Ethical Committee of “Victor Babes” University of Medicine and Pharmacy, Timisoara (No. 25/2011–2014). The primary objective of the study was to evaluate the hypothesis that long-term CPAP therapy may improve blood pressure control in patients with OSA and resistant hypertension (RHTN), and these results were previously published [23]. As a secondary objective, we looked at the effects of CPAP therapy on obesity, as well as the cardiovascular comorbidities. We included patients who had been diagnosed with OSA through polygraphic/polysomnographic analysis, had completed a sleep survey and had a documented medical history of RHTN. The latter was defined as elevated blood pressure with systolic >140 mmHg and/or diastolic >90 mmHg despite simultaneous use of at least three antihypertensive drugs of different classes, including a diuretic [26]. We classified as patients having arrhythmias those with atrial fibrillation and flutter, paroxysmal supraventricular tachycardia (PSVT), ventricular bigeminy/couplets, supraventricular tachycardia (SVT) and sick sinus syndrome. We also included patients with pacemaker or implantable cardioverter defibrillator due to life-threatening arrhythmias. Isolated ventricular or supraventricular beats were regarded as insignificant and not included in the classification. Exclusion criteria were: patients with incomplete data and medical information, as well as incomplete sleep studies, inability or refusal to complete the sleep questionnaires at baseline and follow-up visits, coexistent lung cancer or pregnancy and noncompliance with antihypertensive medication. We also excluded patients that had an identifiable secondary cause of hypertension, other than OSA.

We analyzed the medical records of all the patients who had undergone a sleep study in our lab from 2001 to 2011. Patients with a confirmed diagnosis of OSA and a history of resistant hypertension were invited for an additional clinical review between 2012 and 2014. The patients who were non-compliant with their CPAP treatment served as our control group. Patients were deemed compliant if they had followed the treatment for at least six consecutive months prior to the follow-up evaluation.

At both study visits (baseline and follow-up) all patients had their demographic and clinical data reviewed, including age, sex, weight (kilograms), height (centimeters), neck, waist and hip circumference (centimeters). A history of comorbidities (i.e., cardiovascular disease, peripheral vascular disease, coronary heart disease, heart failure, arrhythmias, diabetes or stroke) and current medication was obtained from patients’ reports and review of available medical documents. Patients also completed a sleep questionnaire (Epworth questionnaire) with eight questions, four response options and a score range between 0 and 24. A score of 10 or more was considered significant for excessive daytime sleepiness.

After the patients had been seated for at least 15 min, one or two readings of systolic and diastolic blood pressure were obtained at a five-min interval using a standard blood pressure manual machine (standard mercury sphygmomanometer FAZZINI, Vimodrone, Italy).

Polysomnography was performed according to standard guidelines [27] using a POLY-MESAM 4 (1998) or STARDUST RESPIRONICS (2005) device. A 32–35 channel polysomnographic recording system (model ALICE 5 RESPIRONICS 2005) was used in selected patients to assess the sleep state and respiratory and cardiac variables. Each automatic recording was reviewed and manually validated by qualified sleep lab personnel.

Hypopneas were defined as ≥50% reduction of airflow in the nasal pressure channel for ≥10 s resulting in an arousal or ≥3% oxygen desaturation. The apnea-hypopnea index (AHI) was defined as the average number of episodes of apnea and hypopnea per hour of objectively measured sleep. The recording montage included electroencephalography, electrooculography and chin electromyography to determine the sleep stages for each 30-s interval. Rib-cage and abdominal respiratory motion sensors along with arterial oxyhemoglobin saturation, oral and nasal airflow and nasal air pressure were used to evaluate episodes of sleep-disordered breathing. A nasal cannula detected nasal airflow, piezoelectric transducers recorded rib-cage and abdominal excursions and a pulse oximeter recorded the oxyhemoglobin saturation and the heart rate.

## 3. Statistical Analysis

Data was stored electronically using Microsoft Excel 2007 and processed using IBM-SPSS, version 18, 2010. Normal distribution was tested with the Kolmogorov-Smirnoff test in scale variables. T-tests were used for comparison of independent samples with normal distribution, Mann-Whitney tests for comparisons of non-parametric distribution groups and chi-square test for comparisons of proportions. For comparisons of repeated measurements, paired t-tests were employed in scale variables and the McNemar test in categorical variables. Agreement of classification was tested with Cohen’s Kappa statistics and interpreted as follows: 0.01–0.20 as none to slight, 0.21–0.40 as fair, 0.41–0.60 as moderate, 0.61–0.80 as substantial, and 0.81–1.00 as almost perfect agreement [28]. For size effect interpretation, partial eta squared (η^2^) or r, as appropriate, were calculated, which were interpreted according to Cohen’s criteria: for η^2^ = 0.01 small effect size, η^2^ = 0.06 medium effect size and η^2^ = 0.14 large effect size and for r: 0.1 to 0.3—small effect; 0.3 to 0.5—intermediate effect; 0.5 and higher—strong effect [29,30].

## 4. Results

### 4.1. Baseline Characteristics

**Figure 1:**
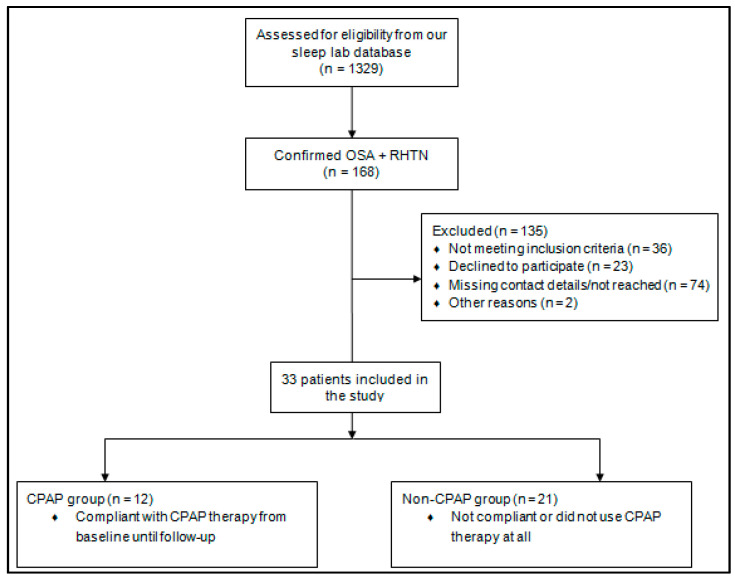
Flowchart of the study design. (OSA—obstructive sleep apnea; RHTN—resistant hypertension; CPAP—continuous positive airway pressure).

We reviewed a total of 1329 patients’ records who had completed their initial visit at our sleep lab between 2001 and 2011. From this group, we identified 168 patients with confirmed sleep apnea and a diagnosis of resistant hypertension. The number meeting the inclusion criteria for our study was 132, of whom 74 had missing contact details or could not be reached, 23 patients declined participation in the study and two patients had died. Follow-up data was collected from the remaining 33 patients with complete evaluations. The patient sample was divided into two groups based on their compliance with CPAP therapy: 12 patients (36.4%)—subsequently named “CPAP group”—followed the CPAP treatment from the diagnosis up until the follow-up visit, while the others were not compliant or did not use CPAP therapy at all (mostly due to reimbursement issues)—subsequently named “non-CPAP group” (Figure 1).

Baseline demographic and clinical characteristics of the patients from the two groups are summarized in Table 1. No significant differences were found between the groups, except for smoking status. The majority of patients in the CPAP group (75%) were never smokers, while in the non-CPAP group the majority of patients (61.9%) were former or active smokers.

The mean follow-up period was 50.9 ± 15.59 months for patients in non-CPAP group and 46.41 ± 21.11 months for patients in the CPAP group.

At baseline, the mean AHI and mean desaturation index (DI) were significantly higher in the CPAP group compared to non-CPAP group (*p* = 0.015 and 0.039, respectively). No other differences were noted between the groups with regards to the somnographic parameters (Table 2).

The classes of antihypertensive drugs used by the patients in the two groups at baseline and follow-up evaluation are listed in Table 3.

### 4.2. CPAP Effect on Change in BMI

The majority of patients included in our study were obese, with a baseline prevalence of obesity at 75% in the CPAP group and 85.7% in non-CPAP group. At the follow-up evaluation, the prevalence of obesity was 83.3% in the CPAP group and 95.2% in non-CPAP group. The shift in prevalence according to McNemar test was not statistically significant in either group (both *p* > 0.05). Further, by calculating the kappa coefficient of agreement for the classification of the severity of obesity between initial and follow-up evaluations in the two groups, we found substantial agreement in CPAP group and moderate agreement in non-CPAP group (Table 4).

**Figure 2:**
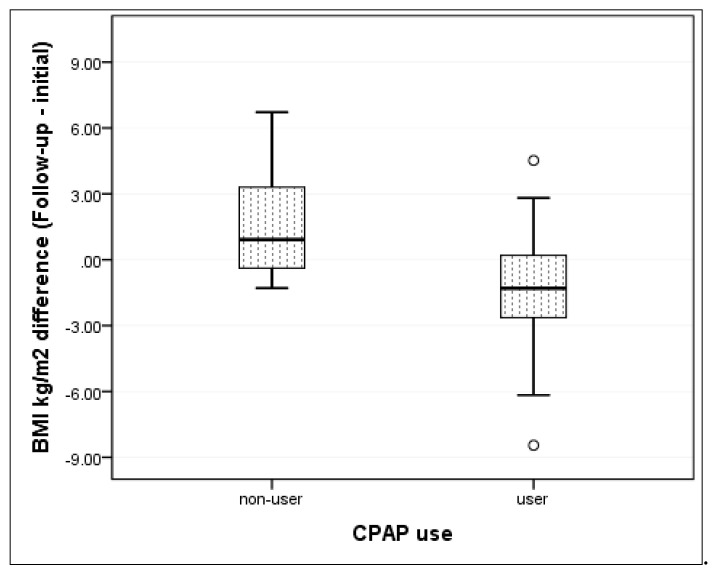
Change from baseline to follow-up in mean BMI according to CPAP usage status. (CPAP—continuous positive airway pressure; BMI—body-mass index; small size circles in the graph represent outliers).

We evaluated the long-term effect of CPAP therapy on weight loss outcomes by looking at the change in anthropometric parameters at the follow-up visit. We observed a significant decrease in BMI in patients using CPAP therapy compared to patients in non-CPAP group (*p* = 0.006), with a large effect size (η^2^ = 0.218). There was an average loss in BMI of 1.4 ± 3.5 kg/m^2^ in patients from the CPAP group, compared to an average gain of 1.6 ± 2.5 kg/m^2^ among the patients not using CPAP therapy (Figure 2).

**Figure 3:**
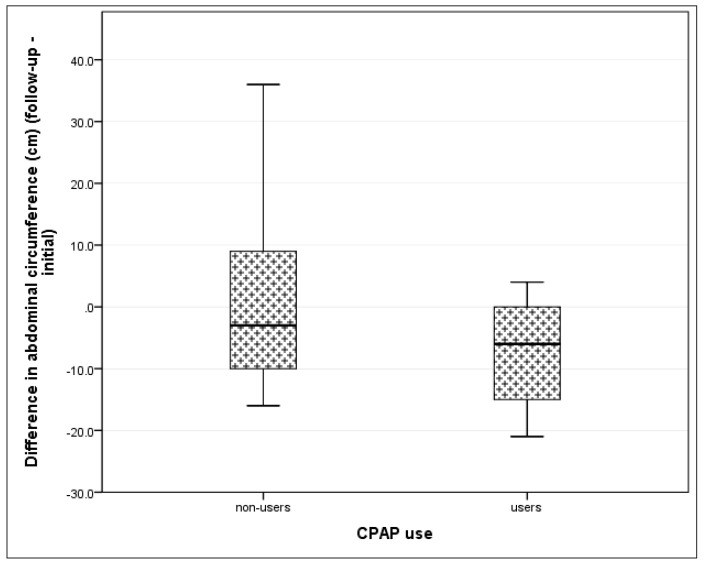
Change from baseline to follow-up in abdominal circumference (cm) according to CPAP usage. (CPAP—continuous positive airway pressure).

We also noted a trend towards improvement in other anthropometric parameters at the follow-up visit in patients from the CPAP group compared to patients from non-CPAP group. The difference in magnitude of change between initial evaluation and follow-up was not statistically significant (Table 5). However, there was a large effect size (η^2^ = 0.444) reduction in mean abdominal circumference in the CPAP group at follow-up compared to baseline evaluation (Figure 3), but no significant change in non-CPAP group.

At the four-year follow-up there was a significant improvement in most polysomnographic parameters in the CPAP group compared to non-CPAP group (Table 6).

### 4.3. CPAP Effect on HR and Prevalence of Cardiovascular Comorbidities

**Figure 4:**
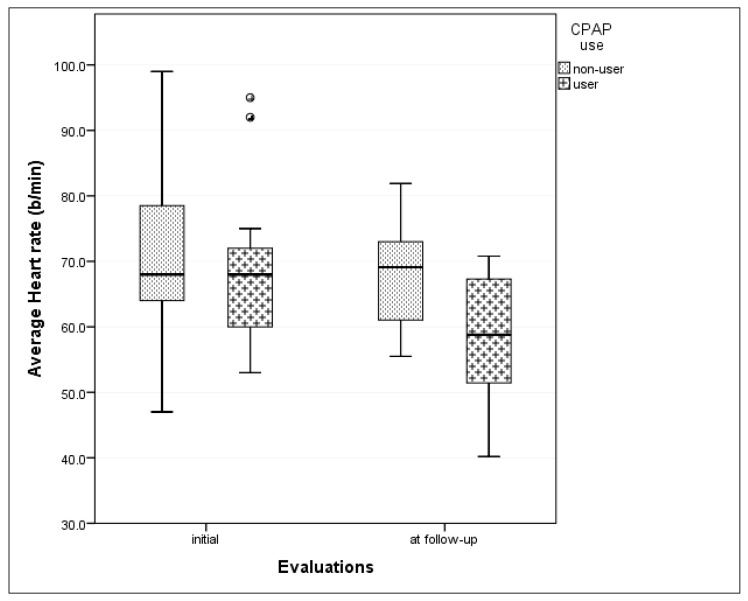
Change in mean heart rate according to CPAP usage between initial evaluation and follow-up. (CPAP—continuous positive airway pressure; small size circles in the graph represent outliers).

The mean heart rate at the follow-up evaluation was significantly lower, with a large effect size (η^2^ = 0.6) in the CPAP-group compared to non-CPAP group. However, there was no statistically significant difference between CPAP use groups in the evolution of mean heart rate between initial and follow-up evaluation (*p* = 0.148) (Figure 4).

Split analyses by CPAP use groups showed non-significant shift in the prevalence of cardiovascular comorbidities between baseline and follow-up evaluation in either group excepting an intermediate size effect (r = 0.37) worsening in the evolution of arrhythmia diagnosis in non-CPAP group (Table 7).

## 5. Discussion

In this observational study we have shown that long-term CPAP therapy may play a role in body weight regulation in patients with OSA and RHTN. Despite the small size of our study sample, we were able to demonstrate a significant reduction in mean BMI in the group of patients who were compliant with the long-term CPAP therapy. This is significant as no specific dietary or lifestyle changes were followed by the patients. Furthermore, there was a significant difference between the groups at baseline with regards to smoking status. The metabolic effects of smoking are known to promote weight loss [31,32], and since the majority of patients in the CPAP group (75%) were never smokers, while the majority of patients in the non-CPAP group (61.9%) were active or former smokers, this is further supportive of our results showing that CPAP therapy may play a role in BMI reduction.

Obesity was present in 81.8% of our study patients, with a mean BMI of 37.03 kg/m^2^ and no significant differences between the two groups in mean BMI or obesity grade, which confirms the hypothesis that obesity and OSA are strongly associated, especially in patients with RHTN. The role of comorbidities in OSA patients has emerged recently, with special attention to cardiovascular and cerebrovascular comorbidities. Some data suggest that CPAP might be protective especially in patients with comorbidities [33]. Recent evidence has also raised questions about the benefits of positive airway pressure therapy in ameliorating comorbidities. One of the priorities for future research in OSA is a definition of subgroups benefitting from CPAP treatment, especially resistant hypertension [34].

A common cause of RHTN is hyperaldosteronism [35] and several studies have shown a link between high plasma aldosterone concentration and the severity of OSA [36,37]. This can, in part, explain the high prevalence of OSA in RHTN patients. In our cohort, no patients with hyperaldosteronism were present among those initially excluded. The majority of patients included in the study were referred to our sleep lab from a cardiology center in order to evaluate the presence of OSA as a secondary cause of RHTN.

Several studies have been published on the presumptive effect of CPAP on body weight in OSA patients, with conflicting results. However, the differences in study design, study population, follow-up period, etc., make the comparison of results difficult.

Two older studies reported positive results of CPAP therapy on weight control. Loube et al. were among the first to describe weight loss associated with CPAP treatment in obese and overweight patients with OSA [38]. Another study [38] that looked at visceral and subcutaneous fat accumulation in OSA patients treated with CPAP found a significant reduction in these parameters after six months of treatment and a significant decrease in serum leptin levels after three–four days of CPAP therapy. The study had an interventional design; however, there was no control group.

Redenius et al. [21] implemented a similar study to ours, that looked at BMI changes after CPAP therapy, with a one year follow-up. The authors reported mixed results, with no significant differences in BMI between treatment and control group at the follow-up evaluation, an increase in BMI in the CPAP group in women and non-obese only and no significant decrease in BMI in any group treated with CPAP. Major differences between this study and ours are the control group consisting of untreated patients only, and a longer follow-up period in our study. In another small interventional study that followed 20 obese patients with OSA for six months, the authors explored the metabolic effects of CPAP therapy. In this study, patients had increased insulin resistance after six months of CPAP treatment and 40% of study patients gained weight significantly [20]. The authors reported a positive correlation between insulin resistance and weight gain; however, there was no control group in this study, while other reports have demonstrated a rapid improvement in insulin sensitivity after two days of CPAP therapy that remained stable for three months [39].

Quan et al. conducted a large randomized controlled trial on the effect of CPAP therapy on weight change, showing that CPAP usage was associated with a modest increase in body weight over a time-frame of six months, with a dose-response type of relationship between hours of adherence and amount of weight gained [40]. In our study we observed a modest decrease in BMI in the treatment group and an increase in BMI in the control group, but over a much longer follow-up period and in a selected population of obese OSA patients associating RHTN. In another large observational cohort study the authors reported no significant change in BMI in the majority of OSA patients treated with CPAP over a follow-up period of five years [41]. It is important to note that the mean BMI at baseline in the last two studies mentioned was significantly lower compared to the mean baseline BMI in our cohort (32.2 and 33.5 versus 37 kg/m^2^). Despite the extensive research on the effect of CPAP therapy on body weight in OSA patients, the novelty of our study consists of selecting a population of OSA patients with heightened cardiovascular risk, associating RHTN, followed-up for a long period of time and having as control a group of OSA patients largely untreated.

There are several mechanisms that could explain our findings, as OSA and obesity are in an interdependence relationship, creating a vicious cycle: while obesity is an important factor in the pathophysiology of OSA, the latter plays a role in further promoting weight gain. Basically, there are two main mechanisms that play a role in weight gain: the negative impact OSA has on energy expenditure and the dysregulation of hunger/satiety hormones. Hormonal changes favor weight gain by stimulating hunger via ghrelin, combined with a low satiety induced by leptin resistance [42,43]. A large systematic review from 2016 explored how CPAP therapy affects the metabolism involved in regulating energy balance [44]. After screening and assessing a number of 42 studies, the authors concluded that while CPAP therapy may reduce energy expenditure it did not induce a compensatory increase in physical activity to counterbalance those reductions. It failed, however, to answer the question as to why changes in body weight occur when alleviating OSA symptoms. Another meta-analysis that included 3181 overweight and obese patients from 25 randomized trials, which had a minimum treatment duration of four weeks and objectively measured BMI [45], tried to answer the question regarding body weight changes with CPAP therapy. The authors of the study concluded that CPAP treatment promoted significant increase in BMI and pointed out the importance of additional therapies for body-weight reduction in obese patients with OSA.

We saw a significant improvement in polysomnographic parameters in patients compliant to CPAP therapy at the four year follow-up. This is of increased significance and further supportive of our results, since the follow-up somnography data was collected without using CPAP therapy for all the patients included in the study.

We did not find significant differences in mean neck, hip or abdominal circumference between the two groups at the follow-up evaluation; however, there was a trend towards improvement in these anthropometric outcomes, with a significant reduction in abdominal circumference in the CPAP group, further supporting the beneficial role of CPAP therapy in promoting weight loss. Studies have shown that neck circumference may be independently associated with HTN, and more so with RHTN due to rostal neck edema [46]. This contributes to the pathogenesis of sleep-disordered breathing by narrowing the upper airway. A large study looking at the effects of CPAP treatment on patients with RHTN revealed that neck size may be a better anthropometric measure than BMI [47].

Long-term CPAP therapy was also associated in our study with a positive effect (i.e., decrease) on the heart rate, objectively measured by nocturnal pulse oximetry, and frequency of arrhythmias at the follow-up evaluation. Atrial fibrillation (AF) is the most common arrhythmia in the general population and has a prevalence of approximately 5% in patients with OSA [48]. There are several mechanisms explaining the frequent association of OSA with AF, among which probably the most important ones are the night time surges in sympathetic tone, as well as the increase in systemic hypertension. Structural changes in the left atrium independently associated with arterial stiffness may further contribute to the increased risk of AF in OSA patients [49]. The arrhythmia prevalence at baseline was numerically higher in the CPAP-group in comparison to non-CPAP group (*p* = 0.274) (Table 7), while at the follow-up evaluation we noticed a decrease in arrhythmia prevalence in the CPAP group (50% vs. 25%) and an increase in non-CPAP group (28.6% vs. 42.9%). As there were no differences between the groups at baseline with regards to age, gender or BMI, and a similar proportion of patients in both groups were on beta-blockers (*p* = 0.938 and *p* = 0.651 at baseline and follow-up, respectively) or calcium channel blockers (*p* = 0.839 and *p* = 0.44 at baseline and follow-up, respectively), the observed decrease in arrhythmia prevalence in the CPAP group may be largely attributed to the effect of long-term CPAP therapy. The baseline difference between the groups in smoking status and severity of OSA may be accountable for the non-significant difference in arrhythmia prevalence at baseline and is further supportive of the positive effect of long term CPAP treatment on the prevalence of arrhythmias. A study by Kanagala et al. has shown that patients with OSA and AF following appropriate CPAP treatment may experience a lower recurrence of atrial fibrillation episodes compared with untreated patients [50]. Our previously published data failed to demonstrate a significant improvement in blood pressure values between CPAP treated and untreated patients with OSA and RHTN; however, blood pressure control was achieved in 75% of patients from the CPAP group [23].

Good adherence to CPAP therapy is of paramount importance in the treatment of OSA. Campos-Rodriguez et al. [51] reported a high adherence rate (74.5%) in RHTN patients after one year of follow-up. What sets our study apart from other retrospective analyses is the cohort of untreated OSA patients which served as a control group. While information regarding CPAP adherence in patients with OSA and RHTN is sparse, we found a relatively high rate of non-compliance to CPAP therapy in the patients included in our study (63.6%). In Romania, this can be easily explained by the fact that CPAP treatment is not reimbursed by the health insurance system, making the treatment inaccessible to many patients, mainly due to high costs. The severity of OSA may play a role in improved CPAP adherence. In our study, the group of patients who were compliant to CPAP therapy had a significant higher AHI and DI at baseline, indicating a more severe disease, compared to patients non-compliant to CPAP treatment. However, the mean values of these two parameters at baseline were in the range of severe OSA for both groups.

Our study has a series of limitations. First, despite the fact that we have identified a potentially larger patient population, only a small number of patients were finally included in the study for the reasons exposed previously. Post-hoc power calculations [52] for the difference of BMI (calculated as initial minus follow-up values) between the two groups indicated a power of 87.5% for this analysis. For the change in heart frequency, calculated as initial minus follow-up values between the two groups, the post-hoc power calculation showed a power of 45%. In order to reach an 80% power, it would have been necessary to have a sample size of 84 patients, 28 in CPAP and 56 in non-CPAP group. Second, the study design was observational and, third, some clinical data were collected based on patients’ reports and may have been biased. Because not all the CPAP machines used by the patients had a data storage medium, the information on the CPAP therapy compliance was limited. However, we were able to send a signal on the potential role of CPAP therapy in promoting weight loss in the subgroup of OSA patients associating RHTN. Further research is warranted in order to clarify the role of CPAP therapy in body weight regulation of patients with OSA and RHTN and thus help refine the approaches in treating these patients.

## 6. Conclusions

In our cohort of OSA patients with RHTN, long-term CPAP use was associated with significant weight loss. Additionally, we noted an improved heart rate and a lower prevalence of arrhythmias in the group of patients compliant to CPAP therapy. Despite the positive effect on body weight and cardiovascular parameters, it is vital that CPAP treatment is accompanied by strong dietary interventions. Future prospective studies focusing on CPAP adherence in high cardiovascular risk patients with OSA may help to evaluate the effect of specific treatment in reducing cardiovascular risk.

## Figures and Tables

**Table 1:** Baseline demographic and anthropometric characteristics according to CPAP usage status at follow-up visit.

Baseline Demographic and Anthropometric Characteristics		Non-CPAP Group (*n* = 21)	CPAP Group (*n* = 12)	*p* Value
Gender	Men no. (%)	11 (52.4)	7 (58.3)	0.741 *
	Women no. (%)	10 (47.6)	5 (41.7)	
Age (years)	Mean (SD)	54.1 (8.3)	55.6 (6)	0.602 **
Smoking status	Never no. (%)	8 (38.1)	9 (75)	**0.041** *
	Former smoker or active smoker no. (%)	13 (61.9)	3 (25)	
Alcohol consumption	No no. (%)	7 (33.3)	5 (41.7)	0.716 *
	Yes no. (%)	14 (66.7)	7 (58.3)	
Systolic blood pressure (mmHg)	Mean (SD)	152.9 (17.9)	147.9 (19.7)	0.468 **
Diastolic blood pressure (mmHg)	Mean (SD)	95.7 (16.3)	95.83 (13.8)	0.983 **
BMI (kg/m^2^)	Mean (SD)	36.4 (5.6)	38.2 (8.9)	0.538 **
Neck circumference (cm)	Mean (SD)	44.8 (3.7)	45.9 (4.9)	0.471 **
Abdominal circumference (cm)	Mean (SD)	121.6 (9.8)	125.9 (17.9)	0.455 **
Hip circumference (cm)	Mean (SD)	119.6 (9.6)	126.9 (20)	0.251 **
Epworth score	Mean (SD)	12.6 (4.6)	11.7 (4.8)	0.578 **

(CPAP—continuous positive airway pressure; SD—standard deviation; BMI—body-mass index). * chi-square test. ** independent samples *t*-test. *p*-values in bold are statistically significant.

**Table 2:** Baseline polysomnographic parameters according to CPAP usage status at the follow-up visit.

Baseline Polysomnographic Parameters		Non-CPAP Group (*n* = 21)	CPAP Group (*n* = 12)	*p* Value
AHI, no. of events/h	Mean (SD)	47.1 (17.9)	65.8 (23.3)	**0.015** *
Desaturation index	Mean (SD)	30.7 (21.9)	51.2 (31.9)	**0.039** *
Mean apnea duration (s)	Mean (SD)	21(4.3)	21.8 (4.1)	0.585 *
Episodes over 5 min with SpO_2_ under 88%	Median (IQR)	0(0)	0(2)	0.326 **
Min SpO_2_ over 2 sec	Mean (SD)	75.3 (12.3)	67.1 (21.8)	0.272 *
Maximum duration SpO_2_ under 88%	Median (IQR)	0.3 (1.1)	0.4 (8)	0.927 **
Average Heart Rate (beats/min)	Mean (SD)	70.7 (13.0)	69.2 (13.5)	0.769 *

(CPAP—continuous positive airway pressure; AHI—apnea-hypopnea index; SD—standard deviation; IQR—interquartile range; SpO_2_—oxygen saturation). * independent samples *t*-test. ** Mann-Whitney test. *p*-values in bold are statistically significant.

**Table 3:** Antihypertensive medication usage between initial and follow-up evaluations.

	Non-CPAP Group (*n* = 21)		CPAP Group (*n* = 12)	
	Baseline	Follow-Up	Baseline	Follow-Up
Alpha blockers, no. (%)	0 (0)	0 (0)	0 (0)	0 (0)
Beta-blockers, no. (%)	15 (71.4)	15 (71.4)	8 (66.7)	7 (58.3)
Calcium channel blockers, no. (%)	13 (61.9)	15 (71.4)	7 (58.3)	7 (58.3)
Diuretics, no. (%)	21 (100)	20 (95)	12 (100)	8 (66.7)
ACE inhibitors, no. (%)	13 (61.9)	9 (42.9)	6 (50)	4 (33.3)
Angiotensin II receptor blockers, no. (%)	10 (47.6)	11 (52.4)	6 (50)	8 (66.7)
Vasodilators, no. (%)	2 (9.5)	0 (0)	1 (8.3)	1 (8.3)
Alpha-2 Receptor Agonists, no. (%)	0 (0)	2 (9.5)	0 (0)	0 (0)

(CPAP—continuous positive airway pressure; ACE—Angiotensin-converting enzyme).

**Table 4:** Agreement of classification of the severity of obesity using BMI categories between initial and follow-up evaluations.

Measurements	CPAP-Group (*n* = 12)	Non-CPAP Group (*n* = 21)
Kappa (coefficient of agreement)	0.664	0.467
Percentage of patients which maintained the class of severity of obesity	75% (9)	61.9% (13)
Percentage of patients which increased severity of obesity	8.3% (1)	23.8% (5)
Percentage of patients which decreased severity of obesity	16.6% (2)	14.3% (3)

(CPAP—continuous positive airway pressure; BMI—body-mass index).

**Table 5:** Evolution of anthropometrical parameters between initial evaluation and follow-up.

Difference between Initial and Follow-Up Evaluation in Anthropometrical Parameters	Non-CPAP Group (*n* = 21)	CPAP Group (*n* = 12)	*p* Value
BMI (kg/m^2^) mean ± SD	−1.6 (±2.5)	1.4 (±3.5)	**0.006** *
Neck circumference (cm) mean ± SD	−0.1 (±2.5)	2.0 (±4.1)	0.078 *
Abdominal circumference (cm) mean ± SD	0.5 (±11.9)	7.3 (±8.6)	0.095 *
Hip circumference (cm) mean ± SD	−0.1 (±26.3)	7.0 (±11.5)	0.385 *

(CPAP—continuous positive airway pressure; SD—standard deviation; BMI—body-mass index). * independent samples *t*-test. *p*-values in bold are statistically significant.

**Table 6:** Evolution of polysomnographic parameters between initial evaluation and follow-up.

Difference between Initial and Follow-up Evaluation in Polysomnographic Parameters	Non-CPAP Group (*n* = 21)	CPAP Group (*n* = 12)	*p* Value
Mean apnea duration (s) (mean ± SD)	−0.6 (±4.8)	2.2 (±5.0)	0.127 *
Episodes over 5 min with SpO_2_ under 88% (mean ± SD)	−1.3 (±3.4)	1.8 (±4.9)	**0.020** *
Min SpO_2_ over 2 sec (mean ± SD)	−3.4 (±13.5)	−13.3 (±20.7)	0.138 *
Maximum duration SpO_2_ under 88% (mean ± SD)	−5.5 (±13.5)	1.7 (±16.7)	0.596 *
Desaturation index (mean ± SD)	−1.6 (±23.0)	30.2 (±31.0)	**0.003** *
Mean desaturation (mean ± SD)	1.4 (±2.7)	−3.0 (±7.4)	**0.027** *
AHI, no. of events/h (mean ± SD)	5.6 (±24.2)	39.5 (±27.9)	**0.001** *
Epworth scale (mean ± SD)	0.4 (±5.1)	4.3 (±6.2)	0.056 *

(CPAP—continuous positive airway pressure; AHI—apnea-hypopnea index; SD—standard deviation; SpO_2_—oxygen saturation). * independent samples *t*-test. *p*-values in bold are statistically significant.

**Table 7:** Baseline and follow-up heart rate and cardiovascular comorbidities.

	Baseline		Follow-Up	
	CPAP-Group (*n* = 12)	Non-CPAP Group (*n* = 21)	CPAP-Group (*n* = 12)	Non-CPAP Group (*n* = 21)
Average HR, beats/min mean (SD)	69.2 (13.5)	70.7 (13.0)	58.6 (9.5)	67.8 (7.8)
Arrhythmias (no./%)	6 (50.0)	6 (28.6)	3 (25)	9 (42.9)
HF (no./%)	4 (33.3)	8 (38.1)	4 (33.3)	11 (52.4)
Stroke (no./%)	2 (16.7)	0 (0)	2 (16.7)	1 (4.8)
CAD (no./%)	8 (66.7)	13 (61.9)	8 (66.7)	18 (85.7)

(HR—heart rate; SD—standard deviation; HF—heart failure; CAD—coronary artery disease).
